# Multicentric Malignant Gastrointestinal Stromal Tumor

**DOI:** 10.4103/1319-3767.45055

**Published:** 2009-01

**Authors:** Shailaja Shukla, Sanjeet K. Singh, Mukta Pujani

**Affiliations:** Department of Pathology, Lady Hardinge Medical College and Smt. Sucheta Kriplani Hospital, New Delhi, India

**Keywords:** GIST, malignant, multicentric

## Abstract

Malignant gastrointestinal stromal tumor (GIST) is a rare type of sarcoma that is found in the digestive system, most often in the wall of the stomach. Multiple GISTs are extremely rare and usually associated with type 1 neurofibromatosis and familial GIST.

We report here a case of a 70-year-old woman who reported pain in the abdomen, loss of appetite, and weight loss for six months. Ultrasound examination showed a small bowel mass along with multiple peritoneal deposits and a mass within the liver. Barium studies were suggestive of a neoplastic pathology of the distal ileum. A differential diagnosis of adenocarcinoma/lymphoma with metastases was entertained. Perioperative findings showed two large growths arising from the jejunum and the distal ileum, along with multiple smaller nodules on the serosal surface and adjoining mesentery of the involved bowel segments. Segmental resection of the involved portions of the intestine was performed. Histopathological features were consistent with those of multicentric malignant GIST-not otherwise specified (GIST-NOS). Follow-up examination three months after surgery showed no evidence of recurrence.

Gastrointestinal stromal tumors (GISTs), formerly classified as leiomyomas or leiomyosarcomas, are mesenchymal tumors of the gastrointestinal tract (GIT). GISTs are now defined as spindle cell, epithelioid, or occasionally, pleomorphic mesenchymal tumors of the gastrointestinal tract, which express the KIT protein (CD117) detected by immunohistochemistry and which lack smooth muscle or Schwann cells as seen in electron microscopy.[1] The term “GIST” is also limited to tumors originating from the pacemaker cells of Cajal located between myenteric plexus cells and smooth muscle cells of the GIT. The immunohistochemical marker c-Kit (CD117) identifies these cells and seems to be the most specific diagnostic marker currently available.[1,2] On ultrastructural examination, the cells of Cajal show both smooth muscle and neural differentiation, accounting for different variants of GIST viz. smooth muscle GIST (myogenic features), gastrointestinal autonomic nerve tumor (GANT with neural features), mixed GIST (both muscle and nerve), or GIST-not otherwise specified (GIST-NOS lacking differentiation). Multiple GISTs are extremely rare, may be familial, and are usually associated with type 1 neurofibromatosis.[3]

## CASE REPORT

A 70 year old woman presented with pain in the abdomen, especially over the right flank and lumbar region, a feeling of mass in the abdomen, and weight loss for six months, with gradual increase in pain and loss of appetite since two months. There were no cutaneous lesions or any familial history.

Clinical examination revealed two irregular, palpable, nonpulsatile, abdominal masses in the upper abdomen measuring 8 × 6 cm and 6 × 5 cm, over the hypogastrium and right hypochondrium-epigastrium region, respectively. Laboratory investigations revealed anemia with hemoglobin (Hb) = 9.0 g%; total and differential leukocyte counts were within normal limits. Biochemistry showed random blood sugar = 114 mg%, urea = 32 mg%, creatinine = 0.8 mg%, total bilirubin = 0.8 mg%, total protein = 5.3 mg%, Alanine Aminotransferase = 31 IU/L, Aspartate Aminotransferase = 42 IU/L, Alkaline Phosphatase = 467 IU/L, and serum. amylase = 97 IU/L.

Ultrasound examination showed a small bowel mass along with multiple peritoneal deposits and a solid mass within the liver. The mass measured 8.5 × 2 cm with a cystic center and a thick echogenic wall. Focal, asymmetrical, circumferential, heterogeneous thickening of the small intestinal wall was also noted. The findings of a barium enema were normal, whereas a barium meal follow-through examination was suggestive of neoplastic pathology of the distal ileum. Fine needle aspiration from the liver mass was inconclusive. The patient underwent exploratory laparotomy and perioperatively, two large growths were seen to arise from the jejunum and distal ileum with multiple smaller nodules in the mesentery; resection of the involved segments of the intestine was done. The liver was also found to be enlarged, raising the suspicion of metastasis, but no biopsy was performed.

### Pathological Findings

#### Gross findings

**Figure 1:**
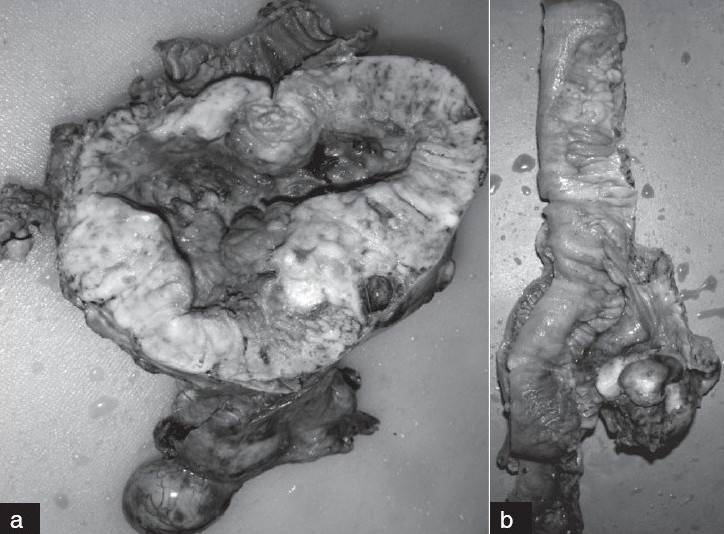
(a) Gross appearance of tumor arising from the wall of jejunum, (b) Tumor in mesentery of ileum

The resected segment of the jejunum measured 8.0 cm in length and 1.8 cm in diameter, along with a large encapsulated, nodular, exophytic mass measuring 13 × 10 × 9 cm arising from the jejunal wall [Figure 1a]. The cut surface showed a gray white-brown, soft-firm tumor with foci of hemorrhage, necrosis, and cystic degeneration. Multiple smaller nodules varying in size from 0.9 to 4.5 cm, soft to firm, friable, and gray-white were found over the serosal surface. The mucosal surface was flattened but was intact.

A large, encapsulated, soft mass measuring 6 × 5 × 5 cm was seen in the mesentery of the ileum [Figure 1b]. Multiple smaller nodules varying in size from 0.2 to 0.6 cm were seen on the serosal surface and in the mesentery, but the mucosal surface was intact.

#### Histological findings

**Figure 2:**
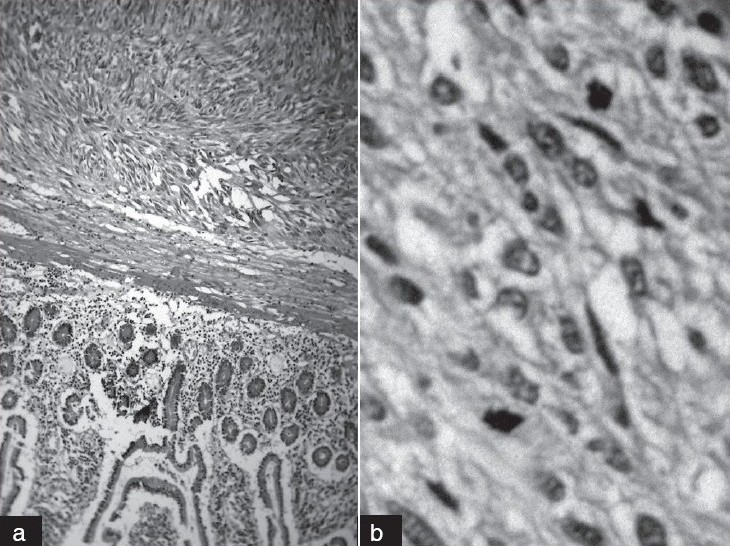
(a) Photomicrograph showing H and E of a highly cellular tumor composed of spindle cells with elongated nuclei pushing muscularis mucosa but not penetrating mucosa, (b) Photomicrograph showing high mitotic figures

Microsections from the jejunal mass showed a highly cellular tumor arising from the muscle layer of the intestine and composed of spindle cells arranged in fascicles [Figure 2a]. The cells had a moderate amount of cytoplasm, and the nuclei were oval to elongated and moderately pleomorphic with high mitotic activity (> 50/50 HPF) [Figure 2b]. The overlying mucosa was intact.

Sections from the mesenteric tumor nodules showed similar histomorphology with pleomorphic nuclei, necrosis, and high mitotic activity (> 50 mitoses per 50 high-power fields).

Immunohistochemical staining was positive for CD34, vimentin, and CD117, weakly positive for smooth muscle actin (SMA), and negative for desmin and S100.

Based on the findings of gross examination, histopathology, and immunohistochemistry, a diagnosis of multiple malignant GIST-NOS was made.

## DISCUSSION

The differential diagnosis of spindle cell tumors of gastrointestinal tract consists of GIST-NOS, leiomyoma, schwannoma, and fibromatosis. Moreover, the scenario becomes complex because of the smooth muscle and/or neural features that can also be found in GISTs. GISTs are rare but are nevertheless the most common mesenchymal neoplasms of the GIT and are thought to arise from the interstitial cells of Cajal—the intestinal pacemaker cells.[2] GIST most commonly arises from the stomach (60–70%) and small intestine (20–30%) with < 10% arising from the rest of the gastrointestinal tract (esophagus, colon, rectum) or extraintestinal sites (omentum, mesentery, retroperitoneum).[1,4,5] In leiomyomas, tumor cells are arranged in fascicles, intersecting each other at right angles. Merging of tumor cells with blood vessel walls is an important clue for diagnosis. Individual cells have elongated, blunt-ended, cigar-shaped nuclei, and acidic, fibrillar cytoplasm. Microscopically, schwannomas show two distinctive patterns: Antoni A areas with spindle cells arranged in a pallisade fashion or an organoid arrangement (verocay bodies), and Antoni B areas in which tumor cells are separated by abundant, edematous fluid.

However, these tumors can be defined better on immunohistochemistry: c-kit is positive only in GISTs; SMA is positive in leiomyomas, fibromatosis, and GISTs (30–40%); desmin is positive in leiomyomas but very rarely in GISTs and fibromatosis; S-100 is negative in fibromatosis and very rarely positive in GISTs or leiomyomas, but positive in schwannomas.

The incidence of GISTs is estimated to be 10–20 cases per million, and they usually occur in patients who are older than 50 years.[1] Intestinal GISTs are more likely to be malignant, as seen in the present case.

Mesenteric GISTs are rare and may arise as primary lesions or as a result of metastatic spread. Usually metastatic, mesenteric GISTs are multiple and may simulate peritoneal carcinomatosis. In the study by Miettinen et al., the average size of mesenteric GISTs was reported to be 16.5 cm.[6]

Clinical symptoms are related to the size and location of the GISTs. Most pathologists use a combination of tumor size and mitotic rate to assess the malignant potential of these tumors. Features favoring malignancy are: size > 5 cm, mitotic rate > 5/50 hpf, high cellularity, nuclear pleomorphism, and necrosis. In general, malignant GISTs are larger, more cellular, and more mitotically active. GISTs that are smaller than 5 cm with ≤ 5 mitoses per 50 consecutive high-power fields are considered to be benign with a low risk for metastasis. Tumors larger than 10 cm with more than five mitoses per 50 high-power fields are considered to be malignant. All tumors falling between these two extremes are considered to be of uncertain malignant potential with intermediate risk for metastasis. Tumors with > 50 mitoses per 50 high-power fields are considered to be highly malignant with an aggressive clinical behavior. The liver and the peritoneum are the most common sites of spread in malignant GISTs.[4]

The immunohistochemical marker, c-Kit (CD117) identifies tumors originating from the pacemaker cells of Cajal and seems to be the most specific diagnostic marker currently available.[1,2]

Omental and mesenteric GISTs are typically positive for CD117, and less consistently, for CD34. They often show alpha-smooth muscle actin reactivity but are virtually negative for desmin and S-100 protein.[6] The present case was positive for CD117, vimentin, and CD34, weakly positive for SMA, and negative for desmin and S100 protein.

GISTs mostly metastasize within the abdomen. In one study of 83 patients, the common site of metastasis was the liver in 38 patients (46%) and the peritoneum in 34 patients (41%).[7] Other sites of metastases include the retroperitoneum, lung, bone, and abdominal scar.

The usual treatment of such tumors is surgical excision, although recently, treatment targeted toward inhibiting the mutant KIT gene by imatinib mesylate, a tyrosine kinase-inhibitor, has also been very successful in recurrent cases.

A recent review noted that patient survival after primary surgical resection of GISTs ranges from 48–80% at five years.[8] For low malignant potential (low-risk) GISTs, the five-year survival rate (approximately 95%) is similar to the normal population, whereas the five-year survival rate ranged from 0 to 30% for high malignant potential (high risk) GISTs, before the introduction of imatinib mesylate. No long-term survival data are available for malignant GISTs on imatinib mesylate therapy. Recurrences are extremely rare for low malignant potential GISTs, while > 80% of high malignant potential GISTs will recur.[8]

Therefore, the treatment of choice for malignant GISTs should be surgical resection, along with imatinib mesylate therapy, and a close follow-up by imaging techniques for recurrences.
